# Gut Microbiota–Medication Interaction in Rheumatic Diseases

**DOI:** 10.3389/fimmu.2021.796865

**Published:** 2021-12-03

**Authors:** Lingshu Zhang, Cong-Qiu Chu

**Affiliations:** ^1^Department of Rheumatology and Immunology, West China Hospital, Sichuan University, Chengdu, China; ^2^Division of Arthritis and Rheumatic Diseases, Oregon Health & Science University, Portland, OR, United States; ^3^Section of Rheumatology, Veterans Affairs (VA) Portland Healthcare System, Portland, OR, United States

**Keywords:** microbiota, disease modifying anti-rheumatic drugs, personalized medicine, biomarkers, prediction and opportunity

## Abstract

Besides its contribution to the development of rheumatic diseases, the gut microbiota interact with anti-rheumatic drugs. The intestinal microbiota can directly metabolize many drugs and indirectly change drug metabolism through a complex multi-dimensional interaction with the host, thus affecting individual response to drug therapy and adverse effects. The focus of the current review is to address recent advances and important progress in our understanding of how the gut microbiota interact with anti-rheumatic drugs and provide perspectives on promoting precision treatment, drug discovery, and better therapy for rheumatic diseases.

## Introduction

Although much of the mechanism is yet to be learnt, current evidence indicates that microbes might be vital environmental factors in initiating and propagating the onset of autoimmune rheumatic diseases. For a long time, specific infectious microorganisms have been suspected to trigger rheumatic diseases in genetically susceptible individuals—for example, *Mycobacterium tuberculosis* was once postulated to cause rheumatoid arthritis (RA), leading to the gold salt remedy to treat the communicable disease (1). This concept was abandoned because of the lack of evidence to prove this was the case. Other bacteria, such as *Porphyromonas gingivalis* (*P. gingivalis*) and *Proteus mirabilis* (*P. mirabilis*), were also considered to be candidate pathogens to cause RA (2–8) as well as *Klebsiella pneumoniae*, *Salmonella*, and *Yersinia* as pathogens for spondyloarthritis (9, 10). However, none of these was proven to directly cause these diseases.

Findings of recent studies rather suggest that dysbacteriosis in microbiome contributes to a range of chronic conditions, such as inflammatory bowel disease (IBD) (11), diabetes (12), multiple sclerosis (13), autism (14), various cancers (15–17), and rheumatic diseases (18).

Dysbacteriosis is the alteration of bacterial composition from healthy status to disease, and this has been well documented in several studies in patients with RA. Vaahtovuo et al. (19) found that, compared with fibromyalgia, patients with early RA had significantly fewer bifidobacteria and bacteria of the Bacteroides-Porphyromonas-Prevotella group, Bacteroides fragilis subgroup, and *Eubacterium rectale*–*Clostridium coccoides* group. Scher et al. (20) reported an expansion of Prevotellaceae but a reduction of Bacteroidaceae in new-onset-RA patients. The disbacteriosis in RA patients was further confirmed and expanded in oral microbiota (21). Interestingly, medically treated arthritic animals in preclinical models and RA patients in clinical remission can restore their gut flora composition (21, 22). These findings highlight the importance of gut microbiota ecological balance to the wellbeing of the host and imply that certain bacteria are beneficial to the host by countering the potential harmful bacteria. On the other hand, these raise the points as to how medications affect the community of microbiota and *vice versa*. The term pharmacomicrobiotics has been introduced for studies investigating the effect of microbiome variations on drug disposition, action, and toxicity (23). This review will focus on our current understanding of the interaction between disease-modifying anti-rheumatic drugs (DMARD) and the gut microbiota.

## How Does Microbiota Influence Rheumatic Diseases?

It is assumed that bacteria first colonize the body of most infants soon after birth. However, several studies reported that bacterial DNA were found in the placenta (24, 25), meconium (26), and amniotic fluid (27). This has put forth an idea that mothers are transferring bacteria to the fetus in the womb before birth to establish a fetal–maternal microbiome relationship. Interestingly, activated memory CD4^+^ T cells developed in fetal circulation (28). The important question raised by these findings is that the placenta is not a totally sterile environment as we thought; the fetus may harbor microbes, which possibly shape our immune system during the earliest days of life even before birth.

The enormous and diverse community of gut microbiota constitutes a distinct network that is vital to make the immune system work functionally, but how the dynamics of microbiome shape autoimmune diseases is unclear. Firstly, the alien microorganisms become the fruitful sources of antigenic variation to interact with immune cells to maintain homeostasis (29). Approximately 70–80% of the immune cells of the body populate in our gastrointestinal tract. As a result of the coevolution, microorganisms and intestinal immune cells form a bidirectional relationship. Multiple autoimmune and inflammatory diseases, such as RA, are classically considered T cell-mediated disorders (30). The microbiota and its metabolite-associated signals are responsible for the activation, polarization, and function of CD4^+^ T cells, including T-bet^+^ T helper type 1 (Th1), GATA3^+^ Th2, retinoic receptor-associated orphan receptor (ROR)-γt^+^ Th17, and FOXP3^+^ T regulatory (Treg) cells (31). A landmark study highlighted that segmented filamentous bacteria (SFB) alone is sufficient to induce the differentiation of lamina propria Th17 cells in mice (32). Notably, mono-colonization with SFB in germ-free mice rapidly induces the onset of autoimmune arthritis and reinstated the lamina propria Th17 cell compartment (33). However, SFB is not found to be colonized in humans. Additionally, a similar sequence between specific microbial peptides and host autoantigens, resulting in the production of cross-reactive T cells targeting both parts, has long been recognized as the molecular mimicry that may be another potential mechanism for the involvement of the microbiome in rheumatic disease (34–36).

Aside from T cells, microbial exposures can activate B cells and induce immunoglobulin (37–40). Antinuclear antibodies (ANA) are a hallmark feature of systemic lupus erythematosus (SLE) (41). Lymphotoxin-deficient mice show the development of ANA by 3 months of age, including anti-U1-ribonucleoprotein, anti-Sm, anti-Scl70/topoisomerase-I, anti-centromere protein B, anti-SSA/Ro52, and anti-Jo1 antibodies. Treating lymphotoxin-deficient mice with antibiotics or in a germ-free condition (42) reduced the prevalence of ANA compared to their littermates (43). Antibodies directed against dsDNA have been associated with SLE disease severity. A recent study reported that serum anti-*Ruminococcus gnavus* strain-restricted antibodies correlated directly with SLE disease activity index (SLEDAI) score and anti-native DNA levels but inversely correlated with C3 and C4 in lupus patients (44). Fecal transplantation from lupus mice to germ-free recipients resulted in enhanced intestinal immune response and upregulated expression of antibody titers against dsDNA (45). Oral gavage of *Roseburia intestinalis* into a mouse model of spontaneous antiphospholipid syndrome in NZW × BXSB F1 triggered the development of anti-human β2 glycoprotein protein I antibodies and thrombotic events (46).

Taking these data together, the above-mentioned studies indicate that commensal microbiota play a role in impacting on the physiological state of the immune cell subsets and are prone to increase the susceptibility to autoimmune responses and reprogramming of immune cells. It must be emphasized that the autoimmunity-triggering effect of these bacteria is the result of a defective counter-mechanism from other commensal microbiota. Prebiotics and probiotics are intended to impact dysbacteriosis and restore the balance between harmful and beneficial bacteria.

## Dialogue Between the Gut Microbiota and the Rheumatic Therapeutics

Since the long-term use of anti-rheumatic drugs and the individual response of patients may vary greatly, the ultimate aim of rheumatologists is to maximize clinical outcomes and minimize their side effects. Most anti-rheumatic drugs are orally administrated and under the process of commensal microorganisms that alter the bioavailability of the drug in the intestine directly or indirectly. With the advent of pharmacomicrobiomics, it has drawn a growing interest to profile interactions between drugs and gut bacteria (47–49).

The metabolism of drugs by trillions of gut microbiota is multidimensional—for example, the prodrug sulfasalazine (SSZ) can hardly be absorbed in the upper intestine, and the majority of this agent is metabolized by the bacterial enzyme azoreductase into its active components sulfapyridine and 5‐aminosalicylic acid (ASA) functionally targeting colon sites (50). The intestinal microbiota harbor diverse β-glucuronidase enzymes that manipulate the pharmacokinetics of oral drugs. Bacteria such as *Clostridium*, *Peptostreptococcus*, and *Staphylococcus* are able to secrete β-glucuronidases (51), which release glucuronic acid (GlcA) sugars from complex carbohydrates. Some chemicals, like nonsteroidal anti-inflammatory drugs (NSAIDs), are conjugated to GlcA (52, 53). Targeting luminal bacterial β-D-glucuronidase can reduce NSAID-related intestinal mucosal injury through halting the hydrolysis of NSAID glucuronides (54).

Aside from the mechanism of microbial enzymes to affect their biotransformation, a recent study emphasized that drug-metabolizing microbial proteins can contribute to the *in vivo* drug metabolism of gnotobiotic mice and provide evidence that metagenomics and genomics sequence data can explain the capacity of both isolated gut bacteria and complete communities to convert specific drugs (55). It was found that, at 7 h after oral gavage of dexamethasone to germ-free mice or gnotobiotic mice mono-colonized with *Clostridium scindens* (*C. scindens*), dexamethasone was significantly reduced and androgen metabolite increased in gnotobiotic mice mono-colonized with *C. scindens* in the caecum. This phenomenon was also found in prednisone, prednisolone, cortisone, and cortisol and demonstrated that *C. scindens* metabolizes endogenous steroid hormones.

A recent study screened 1,197 drugs against 40 representative gut bacterial strains and found that 24% of the drugs affected the *in vitro* growth of bacteria (56). Therefore, the mechanistic understanding of gut microbiota and drugs is still complex; however, manipulating the microbiota in order to promote a better response needs to be further investigated.

## Interaction Between Microbes and Cortisone

Since compound E was introduced by Philip Hench to treat RA successfully (57), glucocorticoids become the strong, broad-spectrum anti-inflammatory and immunosuppressive approach in the art of healing in a range of inflammatory rheumatic diseases (58–60). However, the exact mechanisms of how this first-line therapy impacts on anti-inflammatory pathways are still obscure, and the long-term safety of glucocorticoids is still challenging in rheumatic diseases (59, 61). Recently, a number of corpus have highlighted dysbiotic gut microbiota in SLE (44, 62–64); a reduction of species richness diversity was noted in patients with lupus, with reductions in taxonomic complexity most pronouncedly related to SLEDAI (44). Glucocorticoids are a mainstay therapy to manage flares and remission in SLE (65). The study by Mukherji and co-workers showed that oral gavage with prednisone appeared to have the most significant proportion of *Bacteroidetes* and *Firmicutes* than the control group in MRL/lpr mice. In total, thirty-three bacterial taxa were significantly changed in the prednisone treatment group, and *Rikenella, Mucispirillum, Oscillospira*, and *Bilophila* were of relatively lower abundance at the genera level; *Prevotella* and *Anaerostipes* were enriched as well (66). Additionally, this study also identified that glucocorticoids downregulated *Mucispirillum*, which positively correlated with SLEDAI, and it was previously reported to degrade colonic mucin in the intestines (67). *Oscillospira*, *Rikenella*, and *Bilophila* were positively associated with anti-dsDNA.

Meanwhile, another study screened the gut microbiota in glucocorticoid therapy among patients with SLE. Generally, the observed diversity of bacterial communities was similar between healthy controls and SLE patients with glucocorticoid therapy but statistically different between healthy controls and SLE patients without glucocorticoid therapy. SLE patients treated with glucocorticoids restored the ratio of *Firmicutes* to *Bacteroidetes* and increased a group of core bacteria genera, including *Lactococcus*, *Streptococcu*s, and *Bifidobacterium*, which were reduced in the SLE without glucocorticoids. SLE treated with glucocorticoids reduced activity-related glycan metabolism *via* increasing the abundance of *Bacteroides* in lupus (68).

Taken together, these findings suggest that glucocorticoid therapy has the potential ability to modulate the gut microbiota composition of lupus through some bacteria-based corticosterone synthesis pattern which is still far from clear. Meanwhile, they open up many novel questions and further emphasize the need for novel, more effective treatments for SLE that minimize or eliminate the need for glucocorticoids. Low-dose glucocorticoids are commonly used in combination with other DMARDs to treat RA. How the gut microbiota are affected by glucocorticoids in RA is a subject that is of interest to investigate. Numerous studies have highlighted gut dysbiosis during the different phases of RA, although the variability of results could be subjected to the analysis technology, geographic factors, and clinical progression.

Perturbed microbiome can be normalized after a combination of DMARDs, which may include corticosteroids in RA patients (21). However, it is difficult to dissect how corticosteroids contribute to the collective effects of DMARDs in combination.

## Interaction Between Microbes and Methotrexate

Although originally designed as an anti-cancer therapy, methotrexate (MTX) is now the cornerstone drug for the treatment of various rheumatic diseases and the first-line anchor drug for the treatment of RA over decades (69–71). The possible pharmacological and anti-inflammatory mechanism of this drug is to antagonize folate-dependent processes to suppress the synthesis of purines and pyrimidines, inhibit nuclear factor-κB, Janus kinase signal transducer, and STAT signaling pathway, and promote adenosine signaling (72).

Gastrointestinal side effect is commonly induced by MTX therapy due to the intestinal barrier damage (73–75). Dietary restriction dramatically increased the survival rate of mice exposed to lethal doses of MTX administration. Dietary restriction may suppress intestinal inflammation by upregulating protective intestinal bacteria (*Lactobacillus* genus). However, ablating the gut microbiota through applying broad-spectrum antibiotics eliminates the beneficial effect achieved by dietary restriction. Moreover, administration of probiotic with *Lactobacillus rhamnosus* GG partially mimicked the rescue effect of a dietary restriction (76). Another study indicated that the number of *Bacteroides fragilis* in feces was dramatically decreased in low-dose-MTX-treated mice, while gavage with *B. fragilis* could profoundly ameliorate the MTX-induced inflammatory process (77). In a pharmacokinetic study, a low dose (10 mg/kg) of MTX altered the microbial profile that induced a higher abundance of *Firmicutes* over *Bacteroidetes* and the reverse at high dose (100 mg/kg) in Sprague–Dawley rats. The relative abundance of *Firmicutes* was positively correlated with 2,4-diamino-N-10-methylpteroic acid, which is the MTX degradation produced *via* the excretion of the intestinal bacterial enzyme carboxypeptidase glutamate 2 after MTX treatment at 48 h (78). The microbiota composition also changed after monotherapy with MTX, with a lower abundance of *Enterobacteriales* compared with non-treated patients with RA (79). Zhang *et al*. reported patients with restored RA-related gut and oral microbiome abundance of microbial linkage groups (MLGs) similar to the normal situation after MTX treatment. Enriched gut and oral MLGs also negatively correlated with clinical parameters such as C-reactive protein, anti-citrullinated protein antibodies, and rheumatoid factor (21).

It is well known that MTX response varies among patients with RA, that is, around half of patients fail to achieve an adequately clinical response after MTX therapy (69). A recent study analyzed the gut microbiomes of drug‐naïve, new‐onset-RA patients and observed that the overall bacterial diversity is distinct between MTX responders and non-responders. These non-responders had significantly enriched communities than the responders. A further study revealed a significant increase of MAP-kinase signaling, DNA replication, fatty acid degradation, and ABC transporters in non-responders, as well as a significant decrease of lipopolysaccharide and folate biosynthesis. These data suggest that the human gut microbiota was able to metabolize oral MTX (80). Furthermore, the baseline abundance of gut microbiome features is of great value in predicting treatment outcomes in response to MTX. Notably, a microbiome‐based model by machine learning techniques could suggest a possible future clinical response of the gut microbiome on MTX metabolism (80).

For MTX working as the folate competitive antagonist, folate has been given as an additional medication to reduce the adverse events of MTX, like intestinal toxicity and liver function abnormalities (81). Huang *et al*. reported that leucovorin supplementation not only ameliorated MTX-induced intestinal damage but also remodeled the MTX-induced composition of the bacterial community alternation and increased the abundance of *Bifidobacterium*. Oral gavage of *Bifidobacterium longum* exerts a trophic effect on the intestinal mucosa to ameliorated MTX-induced intestinal damage (82).

The impact of MTX on human gut microbiota has been directly tested using a humanized mouse model (83). Germ-free mice were colonized by stool samples from a healthy human donor. MTX significantly altered the gut microbiota as soon as day 1 of MTX administration, and it lasted for 4 days. A high dose of MTX (50-mg/kg dose for cancer treatment) significantly decreased the Bacteroidetes phylum, while low-dose MTX (1 mg/kg—dose for arthritis treatment) showed the same trend but with a moderate effect. Interestingly, the route of administration of MTX (oral *vs.* intraperitoneal injection) and rescue with folic acid did not significantly affect the overall effect of MTX. The perturbed growth of Bacteriodetes by MTX is confirmed in culture. These findings are reflected in RA patients. Thus, new-onset-RA patients who were responsive to MTX showed a significant decrease in Bacteriodetes relative to those who were not responsive to MTX (80).

**Figure 1:**
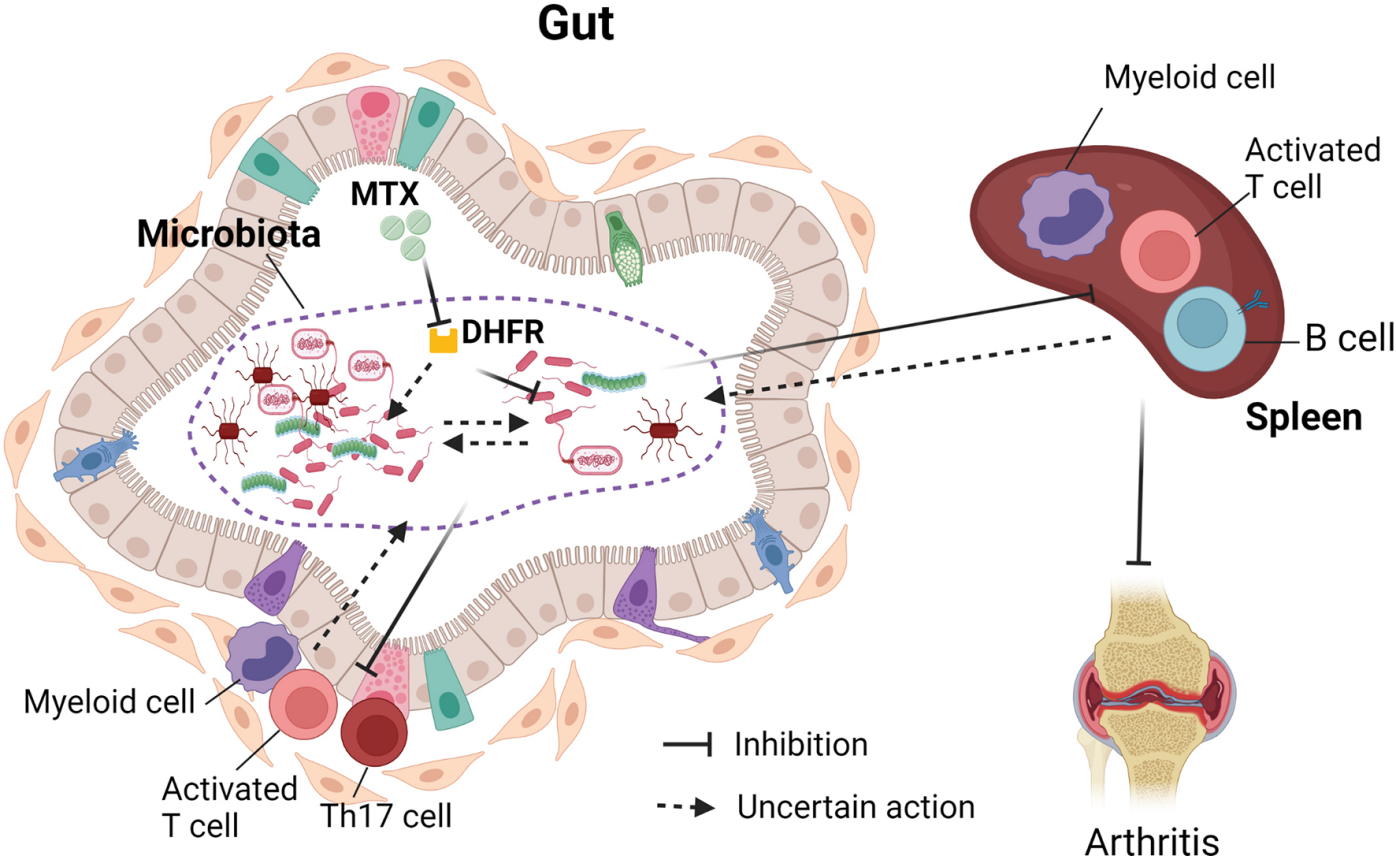
Interaction between gut microbiota and methotrexate (MTX). MTX inhibits bacterial dehydrofolate reductase and affects the growth of bacteria, bacterial transcriptome, and metabolome. There are MTX-sensitive and MTX-resistant bacteria in the human gut. Therefore, MTX treatment affects the community of gut microbiota. The change of gut microbiota post-MTX treatment can suppress immune cells in the periphery and in the intestinal mucosa. It is not clear whether MTX will also affect interactions between gut bacteria and whether the change of immune cellular components will, in turn, affect the gut microbiota.

Interestingly, the microbiota from MTX-treated and MTX-responsive RA patients was able to transfer immunosuppressive effects in gnotobiotic mice. The recipient mice showed a decrease of multiple immune cells, including activated T cells, Th1 cells, B cells, and myeloid cells in the spleen (83). Furthermore, a reduction of activated T cells, Th17 cells, and myeloid cells was also observed in the intestinal mucosa (83). The immunosuppressive effects by MTX-exposed microbiota may be attributed to the different abundance of one phylum (Proteobacteria), 26 genera, and 41 amplicon sequence variants (83). These results suggest that the effects of MTX on microbiota can contribute to the immunosuppressive therapeutic effect of MTX in the hosts. Further investigations are required to delineate how the altered immune cell populations, especially those in the intestinal mucosa, will affect the community of gut flora. The interaction of MTX with the gut microbiota and the effects on host immune activation are illustrated in **Figure 1**.

## Interaction Between Microbes and Sulfasalazine

As *Streptococcus* found in milk was thought as the bacteriological evidence to provoke RA (84), SSZ has been initiated to treat this “infective polyarthritis” since the 1940s (85). Designed as a compound, the most amount of SSZ reaches the colon and is broken into 5‐ASA and sulfapyridine by gut organisms. Although the pharmacological mechanism of action is obscure, SSZ has anti-inflammatory and antibacterial properties to exert beneficial effects on RA, ankylosing spondylitis (AS), and IBD (86, 87).

The administration of antibiotics on germ-free rats showed unchanged SSZ in their caecum and feces. When germ-free rats are infected with four specific bacteria normally found in the intestinal tract of rodents, the rats regain the ability to metabolize SSZ as the conventional rats. These findings suggest that intestinal bacterial metabolism is essential for activating SSZ transformation (88).

Probiotics are “friendly” live microorganisms (bacteria or yeasts) taken as food supplement that promotes favorable benefits for the host by improving the intestinal microbial balance. Co-administration of probiotic strains such as *Lactobacillus acidophilus*, *Bifidobacterium lactis*, and *Streptococcus salivarius* with SSZ modulates azoreductase activity and SSZ metabolism in the colon (89). However, some clinical studies did not claim that the short-term co-administration of probiotics could exert beneficial effects on patients with RA (90) or IBD by changing the metabolism of SSZ. These studies suggest that certain bacterial species possess diazoreductase activity by producing more extensive metabolism of SSZ.

An earlier study showed a significant reduction of the total counts of aerobic bacteria *Escherichia coli* and *Bacteroides* after SSZ therapy and a high frequency of *Bacillus*, but there had been very limited effects on the upper jejunal gastrointestinal flora in patients with RA (91). Treatment with SSZ could alleviate the inflammation and regulate bacterial composition with increasing short-chain fatty acid-producing bacteria (Lachnospiraceae—*Blautia*), lactic acid-producing bacteria (*Lactococcus*), *Mycoplasma*, and decreasing proportions of *Enterococcus* and *Proteobacteria* in 2,4,6-trinitrobenzene sulfonic acid-induced colitis in rats (92).

These results indicate that, in addition to the conversion of SSZ to active drug bacteria in the gut, changes in microbiota composition by SSZ will also contribute to the anti-inflammatory effects of SSZ.

## Interaction Between Microbes and Hydroxychloroquine

Hydroxychloroquine (HCQ) was initially used to prevent and treat malaria and then employed, for its anti-inflammatory properties, to successfully treat various rheumatic diseases, such as SLE, RA, and other inflammatory rheumatic diseases (65, 93, 94).

Previous studies investigated HCQ influence on gut microbiota in Q-fever endocarditis patients. These patients, treated with doxycycline and HCQ, presented significantly lower amounts of *Bacteroidetes* and *Lactobacillus* compared with the controls (95). Systemic rheumatologic conditions are prone to develop more cardiovascular events compared to the general population (96). HCQ administration was reported as a potentially beneficial therapy for K/BxN mice with high-fat diet (HFD) in a mouse model of RA that develops atherosclerosis. HCQ could alleviate the HFD-induced dyslipidemia and atherosclerosis as well as profoundly restored abnormal gut microbiota with a higher abundance of *Akkermansia* and *Parabacteroides* and a lower abundance of *Clostridium sensu stricto* cluster (97). The total glucosides of paeony (TGP) is a traditional Chinese herb medication which has been approved for a variety of rheumatic disease for its anti-inflammatory and immunomodulatory functions (98, 99). The TGP + HCQ group had increased richness of microbiota and had significant changes of *Bacteroidetes* and *Firmicutes* in NOD mice with Sjögren’s syndrome. The proportion of *Lactobacillus* and *Incertae* of phylum *Firmicutes* and *Desulfovibrio* of phylum Proteobacteria was significantly increased, and the abundance of *Bacteroides* and *Alloprevotella* of phylum *Bacteroidetes* and *Pseudoflavonifractor* of phylum *Firmicutes* was significantly decreased in the TGP + HCQ group compared with the control group. The abundance of *Akkermansia* of phylum Verrucomicrobia was significantly decreased in the TGP and TGP + HCQ groups compared with the HCQ group. However, most of these studies do not test the interaction of HCQ alone with gut microbiota alone. Recently, a paper observed that oral gavage of high dose (100 mg/kg) HCQ for 2 weeks significantly increased the relative abundance of phylum *Bacteroidetes*, whereas it decreased that of *Firmicutes* without changing the intestinal integrity and the immunological responses in mice (100).

Investigations into the direct effects of HCQ on gut microbiota will be required to delineate whether HCQ directly impacts bacteria growth or indirectly *via* the immune system of the host.

## Biological Disease-Modifying Anti-rheumatic Drugs

Bioengineered fusion proteins and therapeutic monoclonal antibodies used to treat rheumatic diseases are collectively called disease-modifying anti-rheumatic drugs (bDMARDs). These include agents that inhibit tumor necrosis factor (TNF), interleukin (IL)-1, 6, 17, and 23, T cell co-stimulation, B cell growth factors, and B cell-depleting monoclonal antibody. The targets of these bDMARDs are clearly defined.

TNF inhibitors (TNFi) are the most effective treatments for RA, spondyloarthritis, and IBD after the failure of traditional therapy (101). Ample evidence indicates that TNFi therapy induces mucosal healing and restores gut microbiota dysbiosis in clinical and experimental models (102, 103). However, the interaction between gut microbiota in patients with rheumatic disorders and TNFi is not thoroughly investigated. Etanercept (ETN) therapy showed major intestinal composition changes compared with treatment-naïve RA patients who possessed more abundant *Lactobacillus* as reported before (21, 104). Patients under treatment with ETN present enriched *Cyanobacteria*, while *Deltaproteobacteria* and *Clostridiaceae* were decreased than in treatment-naïve patients (79). Cyanobacteria produce a source of novel bioactive secondary metabolites that may help to modulate the immune system and result in attenuating RA (105, 106).

The gut microbiota is always considered as a vital environmental factor in triggering AS (107). ETN therapy markedly reduced the incidence, arthritis progression, and inflammatory cytokines, such as TNF and IL-17A, in the serum, recovered intestinal barrier function as well as restored the gut microbiota composition similar to that in naïve mice in a proteoglycan‐induced AS model (108). A recent study observed that TNFi treatment had better improvement in AS nonsmokers than in AS smokers. The relative abundance of the microbiota is more prone to be increased in AS nonsmokers after treatment with TNFi for 6 months. In addition, some bacteria, including *Actinomyces*, *Agathobacter*, *Bilophila*, *Klebsiella*, *Lachnospiraceae*_*NK4A136*, *Ruminococcaceae*-*UCG*- *002*, and *Ruminococcaceae*_*UCG*-*005*, were sensitive to TNFi treatment in AS nonsmokers, while *Bacteroides*, *Faecalibacterium*, *Lachnoclostridium*, *Parabacteroides*, *Blautia*, *Butyricicoccus*, and *Escherichia*-*Shigella* were not. This suggests that these bacteria were tolerant to TNFi treatment (109). Since TNFi do not work for all patients, one challenge to clinicians is to investigate the biomarker that can predict the clinical response to TNFi. Another recent study in patients with spondyloarthritis treated by TNFi (most of which are ETN) revealed no significant modification of a particular taxa after 3 months of treatment. It should be noted that the responder patients showed only few mild differences in microbiota composition at order level than in non-responder patients. Interestingly, a higher proportion of the *Burkholderiales* order before TNFi treatment was strongly correlated with the responding patients after 3 months of treatment, suggesting that certain intestinal bacteria can possibly predict the clinical response as a biomarker for TNFi efficacy in patients with spondyloarthritis (110). In Crohn’s disease, infliximab non-responders had a higher abundance of baseline *Blautia, Faecalibacterium, Roseburia*, and *Negativibacillus* genera, while a higher abundance of baseline *Hungatella, Ruminococcus gnavus*, and *Parvimonas* was found in infliximab responders (111). Clearly, more studies including a large number of patients are required to replicate the findings in these studies before profiling of microbiota as a biomarker for predicting response to TNFi can be applied in clinical practice.

## Concluding Remarks

Over a decade of intensive work on the biological activity of gut microbiota spurs inspired enthusiasm to explore the involvement of our resident bacteria in immune processes of the host. There is ample evidence highlighting that gut microbiota interact extensively with anti-rheumatic drugs. In addition to the well-known effect of bacteria on the conversion of inactive prodrugs to active drug, we now learned that DMARDs, such as MTX, can directly affect the growth of gut flora. Furthermore, alteration of the gut microbiota may also contribute to the immunosuppressive effects of MTX. Clearly, further studies are required to identify microbiota which can mediate immune suppression in the host. The other clinically relevant aspect of the interaction of microbiota with DMRADs is towards personalized medicine. Identifying unique individual gut microbial signature may help clinicians to choose a most likely responsive drug for the patient and one devoid of adverse effects.

## Author Contributions

All authors listed have made a substantial, direct, and intellectual contribution to the work and approved it for publication.

## Funding

The work of LZ is supported by the Scientific Application and Foundation Project of Science and Technology, Department of Sichuan Province (no. 2020YJ0021), and the Scientific Research Project of Health Commission of Sichuan Province (no. 20PJ050). The work of CQC is supported by an innovative award from the American College of Rheumatology Research Foundation and by a VA Merit Review grant (I01BX005195).

## Conflict of Interest

The authors declare that the research was conducted in the absence of any commercial or financial relationships that could be construed as a potential conflict of interest.

## Publisher’s Note

All claims expressed in this article are solely those of the authors and do not necessarily represent those of their affiliated organizations, or those of the publisher, the editors and the reviewers. Any product that may be evaluated in this article, or claim that may be made by its manufacturer, is not guaranteed or endorsed by the publisher.
